# Functional nanovesicles displaying anti-PD-L1 antibodies for programmed photoimmunotherapy

**DOI:** 10.1186/s12951-022-01266-3

**Published:** 2022-02-02

**Authors:** Hu Chen, Pengfei Zhang, Yesi Shi, Chao Liu, Qianqian Zhou, Yun Zeng, Hongwei Cheng, Qixuan Dai, Xing Gao, Xiaoyong Wang, Gang Liu

**Affiliations:** 1 State Key Laboratory of Molecular Vaccinology and Molecular, Diagnostics & Center for Molecular Imaging and Translational Medicine, School of Public Health, Xiamen University, Xiamen, 361102 China; 2 Institute of Molecular Immunology, School of Laboratory Medicine and Biotechnology, Southern Medical University, Guangzhou, 510080 China; 3 Tongren Hospital, Shanghai Jiao Tong University School of Medicine, Shanghai, 200336 China

**Keywords:** Photoimmunotherapy, Anti-PD-L1 antibodies, Targeting delivery, Nanovesicles

## Abstract

**Background:**

Photoimmunotherapy is one of the most promising strategies in tumor immunotherapies, but targeted delivery of photosensitizers and adjuvants to tumors remains a major challenge. Here, as a proof of concept, we describe bone marrow mesenchymal stem cell-derived nanovesicles (NVs) displaying anti-PD-L1 antibodies (aPD-L1) that were genetically engineered for targeted drug delivery.

**Results:**

The high affinity and specificity between aPD-L1 and tumor cells allow aPD-L1 NVs to selectively deliver photosensitizers to cancer tissues and exert potent directed photothermal ablation. The tumor immune microenvironment was programmed via ablation, and the model antigen ovalbumin (OVA) was designed to fuse with aPD-L1. The corresponding membrane vesicles were then extracted as an antigen–antibody integrator (AAI). AAI can work as a nanovaccine with the immune adjuvant R837 encapsulated. This in turn can directly stimulate dendritic cells (DCs) to boast the body's immune response to residual lesions.

**Conclusions:**

aPD-L1 NV-based photoimmunotherapy significantly improves the efficacy of photothermal ablation and synergistically enhances subsequent immune activation. This study describes a promising strategy for developing ligand-targeted and personalized cancer photoimmunotherapy.

**Graphic Abstract:**

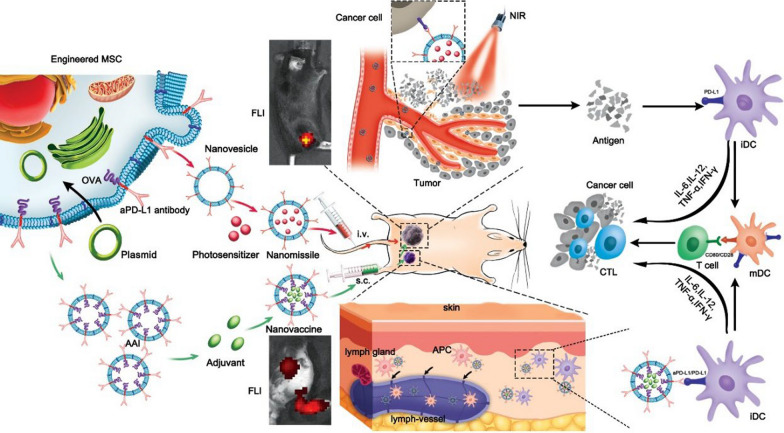

**Supplementary Information:**

The online version contains supplementary material available at 10.1186/s12951-022-01266-3.

## Introduction

Immunotherapy is a novel strategy for tumor therapy that works via an activated immune system [1–3]. However, the tumor microenvironment is immunosuppressive, and conventional delivery of immune adjuvants and tumor-related antigens (TAAs) cannot effectively activate the tumor immune response [4–6]. There is low-infiltration of antigen-specific CD8 + cytotoxic T lymphocytes (CTLs) when the antigens or adjuvants are simply injected into the skin, thus leading to a limited therapeutic effects [7]. Fortunately, TAAs or immunogenic fragments of cancer cells released during tumor apoptosis can be phagocytosed by infiltrating antigen presenting cells (APCs); this in turn can activate the body's immune response to the tumor [8, 9].

Photoimmunotherapy has shown value versus conventional immunotherapies. Recent studies have shown that photothermal-based immunotherapy can induce tumor cell death via high temperatures while simultaneously activating the tumor immune response [10–16]. The damaged cancer cell fragments can be phagocytosed and presented by dendritic cells (DCs) to then activate antigen-specific T-cells. Meanwhile, the apoptotic cancer cells release large amounts of TAAs and cytokines such as IFN-γ that can upregulate PD-L1 expression of tumor cells and DCs. These may reversely inhibit CTL function or exhaust T cells [17, 18]. Moreover, a lack of effective photosensitizers, adjuvants, or delivery carriers to cancer sites can hamper further development. Thus, a versatile nanoplatform to selectively deliver photosensitizers or adjuvants to target specific cell populations and tumor tissues while overcoming the above-mentioned challenges would have significant value.

Actively targeting nanoplatforms to the tumor microenvironment often requires conjugation of targeting ligands. Chemical covalent linkage of receptors or ligands to deliver photosensitizers or adjuvants may cause a loss of tumor targeting and lead to limited photoimmunotherapy. Antibodies can facilitate immunoregulation and binding to specific markers on tumor cells [19–21]. Naturally expressed antibodies or targeting peptides can maintain their biological activity facilitating selective delivery of photosensitizers or adjuvants to antigen-expressing tumor cells or DCs [22, 23].

Cell membrane-derived nanovesicles (NVs) are biogenic nanocapsule structures obtained by crushing or squeezing selected cell membranes [24–27]. NVs display functional proteins on the membrane and can be prepared by modifying cells with gene editing technology [28–30]. Previous studies have shown that PD-1 receptor displayed on NVs with immune checkpoint inhibitory activity can act as a cancer nanovaccines [31–33]. Vesicular antibodies (VAs) displaying hGC33/KM3934 antibodies have also been reported and can specifically bind to tumor cells for targeted drug delivery; they can also recruit natural killer cells for tumor removal [34]. Modified NVs can simultaneously express antiPD-L1 antibodies (aPD-L1) and be a promising drug delivery vehicle for targeting PD-L1 ligand-overexpressed cancer cell and APCs [35–37].

**Fig. 1:**
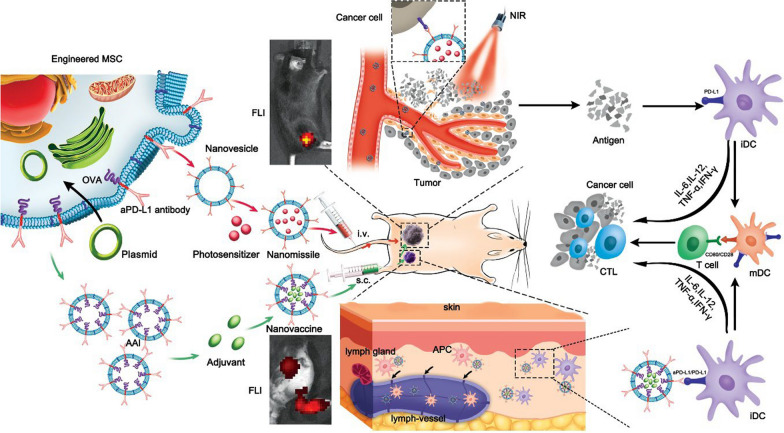
Functional nanovesicles displaying anti-PD-L1 antibodies for programmed photoimmunotherapy. Primarily, aPD-L1 and OVA were expressed on the membrane of MSCs through genetic modification. Afterwards, two different cytomembrane-derived nanovesicles were extracted and loaded with photosensitizer (ICG) and immune adjuvant (R837) by ultrasound, respectively. Then, photosensitizer was delivered to the tumor area via the targeting effect of aPD-L1 after aPD-L1 NVs-ICG was injected through the tail vein. Following thermal ablation of the primary tumor was caused under laser irradiation, inducing the tumor immune microenvironment was reprogrammed. After AAI-R837 were subcutaneous immunization, adjuvant and OVA were orientated delivered to DC though aPD-L1, which can boost the value-added differentiation of CTL

To optimize the curative effect of malignant tumors, we describe here a novel method based on cell membrane-derived biomimetic nanovesicles displaying aPD-L1 for photoimmunotherapy (Fig. 1). Bone marrow mesenchymal stem cells (MSCs) with low immunogenicity were engineered to express aPD-L1 onto the membrane for preparing biogenic nanovesicles (aPD-L1 NVs) as a targeted delivery vector. Using the specific binding of aPD-L1 and PD-L1 ligands, aPD-L1 NVs were loaded with the photosensitizer indocyanine green (ICG) and specifically enriched in the tumor microenvironment for directed photothermal ablation.

The tumor immune microenvironment was reprogrammed via PTT to induce release of cytokines such as IL-6, IL-12 and IFN-γ, invasion of immune cells (such as DCs and CTLs), and regulation of the immune checkpoint. Thus, it is an immunotherapy for residual and distal lesions. Accordingly, the model antigen ovalbumin (OVA) served as a tumor-associated antigen and was designed to be co-expressed with aPD-L1 on the membrane of MSCs and the corresponding membrane vesicles. OVA was extracted as an antigen–antibody integrator (AAI) and is a promising multifunctional nanoplatform. With immune adjuvant (R837) encapsulated in AAI (AAI-R837), antigens and immune adjuvants are delivered to antigen-presenting cells (APCs) in a spatio-temporal way for follow-up immunotherapies. AAI can be a carrier for targeted delivery and can also carry antigen signal to APCs to promote the mature differentiation of DCs, thus boosting the immune response to tumors. Afterwards, AAI-R837 can work as a nanovaccine to facilitate immunotherapy. In this treatment strategy, the multi-functional nanoplatform was used in a combined way for photoimmunotherapy. The material achieved satisfactory inhibitory effects on malignant melanomas.

## Results and discussion

### Preparation and characterization of the aPD-L1 NVs

**Fig. 2:**
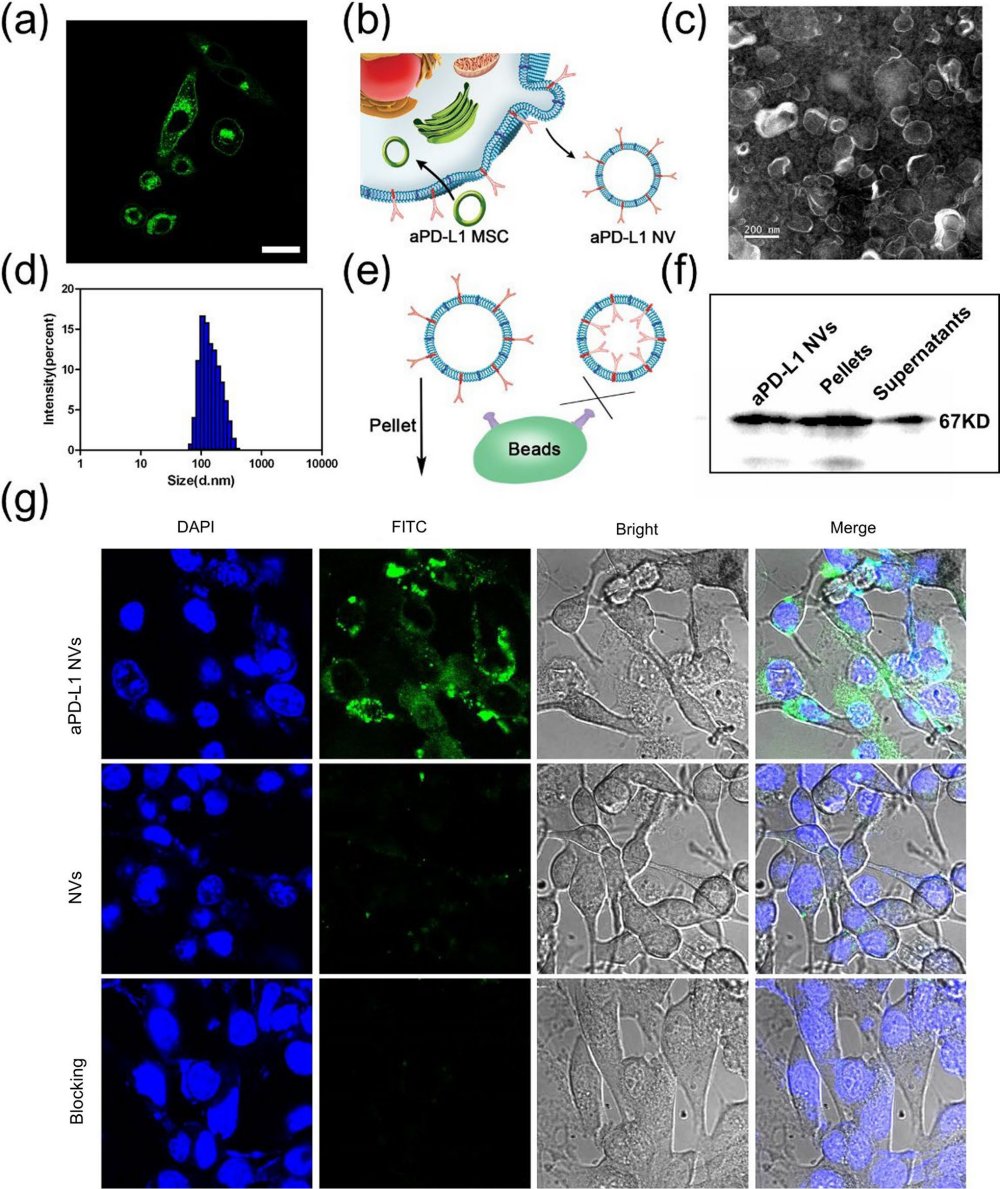
Preparation and characterization of the aPD-L1 NVs. **a** Establishment of aPD-L1 expressed on MSCs’ membranes by immunofluorescence. Scale bar: 50 µm. **b** Schematic diagram of aPD-L1 displayed MSC and aPD-L1 NV extraction. **c**TEM images for aPD-L1 NVs negatively stained with uranyl acetate. Scale bar: 200 nm. **d** Dynamic diameter of aPD-L1 NVs determined by the dynamic light scattering (DLS). **e** Schematic diagram of the CO-IP assay principle. **f** Existence of aPD-L1 on the NVs were examined by Co-IP assay. **g** CLSM image to show the ability of aPD-L1 NVs to specifically target binding with B16-F10 cells. aPD-L1 NVs were labeled with FITC and nuclei was labeled with DAPI. Scale bar: 50 µm

A plasmid containing aPD-L1 fragments was first constructed to prepare aPD-L1 displaying MSCs. Then a lentiviral vector was used to infect MSCs extracted from the bone marrow cavity of C57BL/6 embryonic mice with the aPD-L1 displayed on the outside of the cell membrane (Fig. 2b). Subsequently, the expression of aPD-L1 on the MSCs membrane was verified by immunofluorescence (Fig. 2a). aPD-L1 NVs made from engineered MSC membranes were obtained via ultrasonication. Afterwards, the morphology of negatively stained aPD-L1 NVs was observed in transmission electron microscopy (TEM) (Fig. 2c). The diameter of aPD-L1 NVs was determined to be 100 to 150 nm via dynamic light scattering (DLS) (Fig. 2d). Furthermore, co-immunoprecipitation (CO-IP) assay and Western blot (WB) analysis were employed to detect the existence of aPD-L1 on the NVs (Fig. 2f). Antibodies were indeed expressed on the outer layer of the aPD-L1 NVs. Figure 2e shows a schematic diagram of the CO-IP assay principle.

To test the binding ability of aPD-L1 NVs to the B16F10 melanoma cells, NVs and aPD-L1 NVs labeling fluorescent dye FITC were incubated with B16F10 cells for 3 h and then imaged with confocal laser scanning microscopy (CLSM). Significant fluorescence was observed in the periphery of B16F10 cells after being incubated with FITC-aPD-L1 NVs: They were five-fold brighter than FITC-NVs (Fig. 2g). These data indicate that the aPD-L1 NVs have a better ability to anchor to the cell membrane of B16F10 cells than NVs without aPD-L1. FITC-aPD-L1 NVs were re-incubated with B16F10 cells for 3 h after the PD-L1 ligand on B16F10 cells was blocked with aPD-L1. At the indicated time, less fluorescence signal was observed on the periphery of B16F10 cells via CLSM (Fig. 2g). The results demonstrated that the aPD-L1 NVs can specifically bind to B16F10 cells via interactions between aPD-L1 with PD-L1 ligand.

### Preparation and characterization of nanomissiles (aPD-L1 NVs-ICG)

**Fig. 3:**
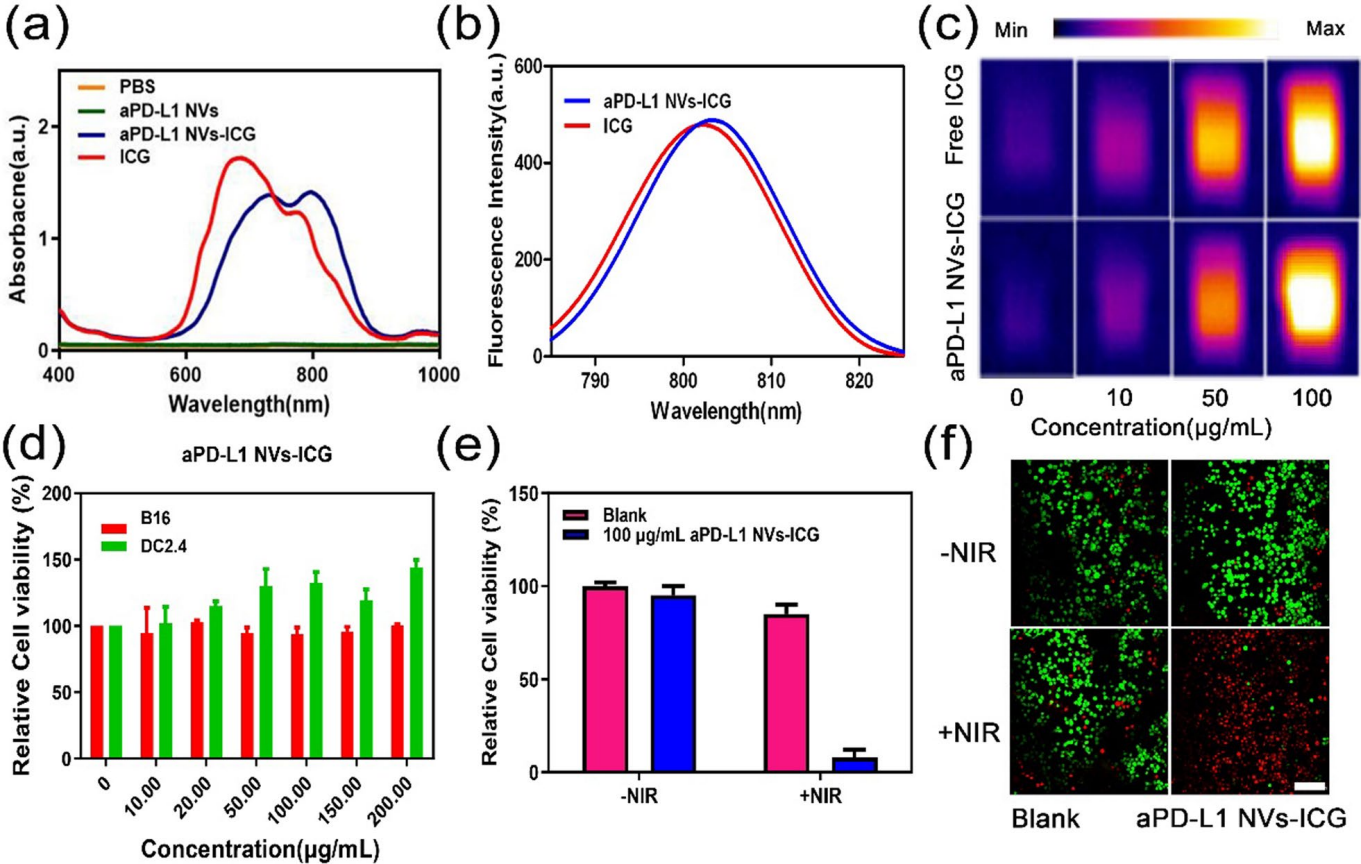
Preparation and characterization of nanomissiles (aPD-L1 NVs-ICG). **a** Vis–NIR absorbance spectra of PBS, aPD-L1 NVs, ICG and aPD-L1 NVs-ICG. **b** Fluorescence spectrum of ICG and aPD-L1 NVs-ICG at 20 μg/mL. **c** IR images of the aPD-L1 NVs-ICG and ICG with corresponding concentration under the irradiation of 808 nm laser (1 W/cm^2^, 5 min). **d** Cell viability of the different concentrations of aPD-L1 NVs-ICG on B16 cells and DCs after co-incubation for 24 h. **e** Photothermal treatment effect of the B16 cells with aPD-L1 NVs-ICG with or without 808 nm laser irradiation (1 W/ cm^2^, 5 min) in vitro. **f** Living and dead cell staining images of the B16 cells treated with aPD-L1 NVs-ICG with or without 808 nm laser irradiation (1 W/cm^2^, 5 min). Living cells were stained with calcein acetoxymethyl ester (AM, green fluorescence) and dead cells were stained with propidium iodide (PI, red fluorescence) dye. Scale bar: 100 μm

The biogenic nano-vector has excellent biocompatibility, and the MSCs used here have a low immunogenicity; thus, they are not easily cleared by the immune system and can extend the circulation time in vivo [38]. The aPD-L1 NVs were used as a carrier for indocyanine green (ICG, aPD-L1 NVs-ICG) after loading via ultrasonication. Ultraviolet (UV) absorption spectrum showed that both aPD-L1 NVs-ICG and free ICG had the highest absorption at about 800 nm (Fig. 3a) indicating successful encapsulation of ICG in the NVs; they have the same UV absorption capacity. The loading efficiency of ICG was further evaluated by UV absorption. The encapsulation rates of ICG were defined as 80% (Additional file 1: Figure S1a, c). Fluorescence spectra indicated that aPD-L1 NVs-ICG had the same fluorescence properties as free ICG (Fig. 3b). To verify the photothermal performance of aPD-L1 NVs-ICG in vitro, a near-infrared (NIR) laser at 808 nm (1 W/cm^2^, 5 min) was used to irradiate aPD-L1 NVs-ICG and free ICG at different concentrations; real-time detection used a NIR thermal imager (Fig. 3c). The results demonstrated that aPD-L1 NVs-ICG had the same photothermal properties as free ICG in equal concentrations. Importantly, when the concentration of aPD-L1 NVs-ICG was 100 μg/mL, the temperature reached 50 ℃ and could kill cancer cells (Additional file 1: Figure S2). Therefore, aPD-L1 NVs-ICG can be used to as a nanomissile for photothermal therapy.

Subsequently, a standard methyl thiazolyl tetrazolium (MTT) test was used to verify the safety of different concentrations of aPD-L1 NVs-ICG on DC2.4 cells (a representative normal cell) and B16F10 melanoma cells (a representative cancer cell). The aPD-L1 NVs-ICG have no obvious toxic effects on DC2.4 cells and B16F10 melanoma cells (Fig. 3d). In addition, we verified the photothermal treatment effect of aPD-L1 NVs-ICG at 100 μg/mL on cancer cells in vitro. Cell viability was measured via a MTT assay after 808-nm laser irradiation (1 W/cm^2^, 5 min) was implemented on the B16F10 melanoma cells with or without aPD-L1 NVs-ICG. The aPD-L1 NVs-ICG had good photothermal killing effects on B16F10 melanoma cells (Fig. 3e). The survival of cancer cells after photothermal treatment was directly observed under CLSM after being stained with calcein acetoxymethyl ester (AM) and propidium iodide (PI) dye (Fig. 3f).

### Biological distribution and PTT of nanomissiles (aPD-L1 NVs-ICG) on tumors in vivo

Inflammation-induced PD-L1 propagates expression in the tumor microenvironment, thus inhibiting the antitumor cytotoxic T-cell response [39–41]. Therefore, the nanovesicles displaying aPD-L1 can not only passively target the tumor site through the EPR effect, but can also accumulate at the tumor site through active targeting effect of aPD-L1 and PD-L1 receptor-specific binding. To verify this effect, free ICG, NVs-ICG, and aPD-L1 NVs-ICG were injected intravenously into C57BL/6 mice with subcutaneous melanoma.

**Fig. 4:**
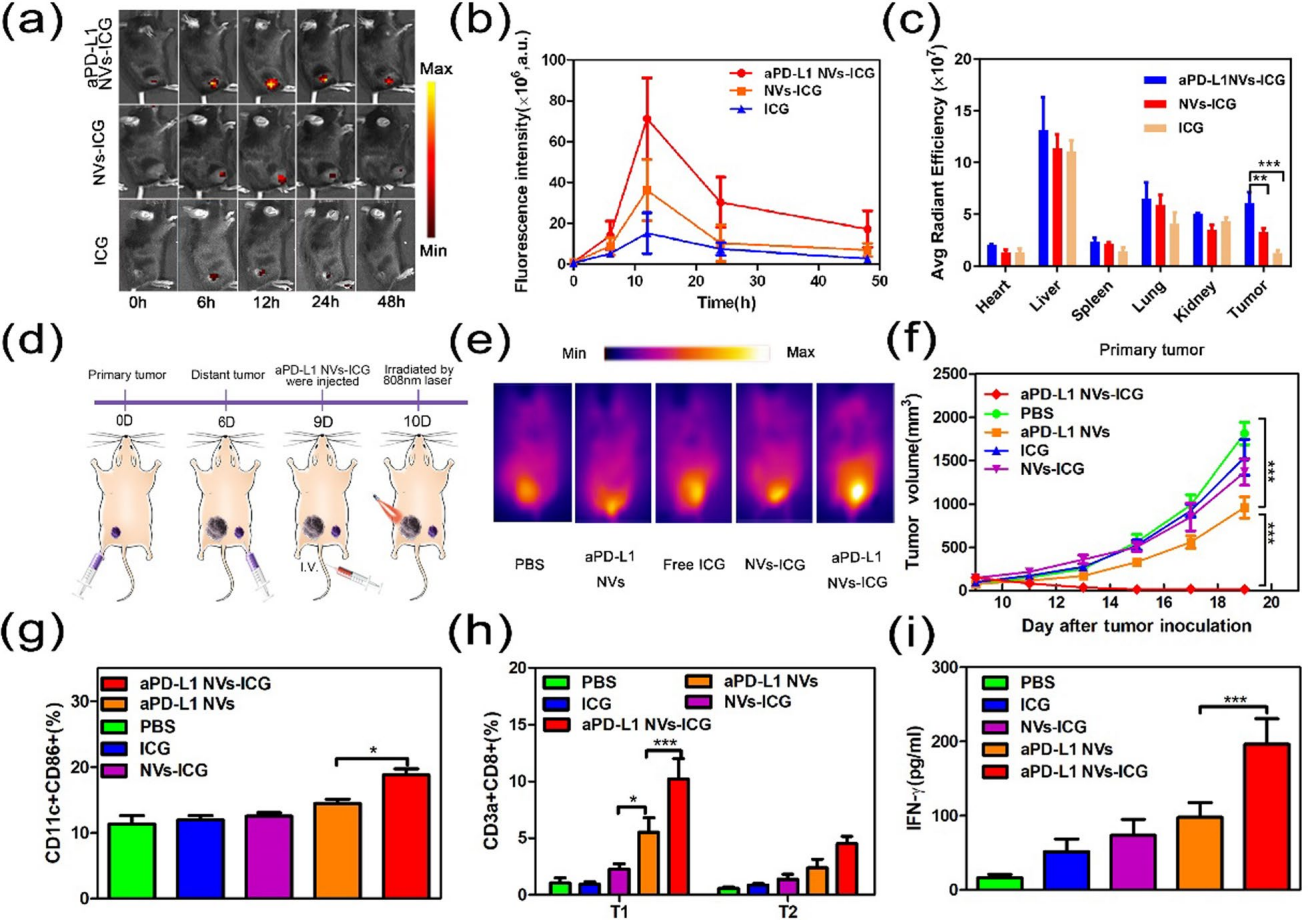
Biological distribution and PTT of nanomissiles (aPD-L1 NVs-ICG) on tumor in vivo. **a** FL images of B16-F10 tumor-burdened mice at different points in time after injection of different drugs through tail vein. **b** The curve of fluorescence signal intensity in tumor area with time. **c**Fluorescence signal intensity of major organs and tumors of the mice in **a** at 48 h. **d** Schematic diagram of photothermal treatment for double tumor-bearing mice. **e** NIR image of the tumor after different treatments under 808 nm laser irradiation (0.75 W/cm^2^, 8 min). **f** Tumor volume growth curve for irradiated primary tumors (in left flank) in mice after PTT with different drugs. **g** The content of mature DC cells in the lymph nodes of mice was detected via analysis of dendritic cell maturation markers (CD11c + CD86 +) by flow cytometry after PTT for 3d. **h** The content of CD8 + T cells in bilateral tumors of mice was detected via analysis of CD8 + T cells markers (CD3a + CD8 +) by flow cytometry after PTT for 3d. T1 represents the primary tumor at the left posterior back and T2 represents the distal tumor at the right posterior back. **i** Production of IFN-γ in serum from the mouse determined by ELISA after treated with PTT for 3d. n = 3 ~ 5. P values were calculated by Student's t test (***P < 0.001, **P < 0.01 or *P < 0.05)

The drug distribution in vivo was monitored by an IVIS Lumina II imaging system. After 12 h and 24 h of administration, the aPD-L1 NVs-ICG had a significant fluorescence signal at the tumor site, and metabolites changed more slowly in vivo than free ICG and NVs-ICG (Fig. 4a, b). To further observe the drug distribution in the organs, mice were euthanized at 48 h, and the heart, liver, spleen, lung, kidney, and tumor tissue were acquired for detecting fluorescent signal. Significant fluorescent signal was clearly observed in the tumor site treated with aPD-L1 NVs-ICG; most of NVs-ICG and free ICG remained in the liver tissue (Fig. 4c and Additional file 1: Figure S3). The imaging data and distribution of aPD-L1 NVs-ICG in vivo confirmed better tumor accumulation.

The efficacy of aPD-L1 NVs-ICG as a photothermal therapy was investigated next. A double melanoma model was established in C57BL/6 mice through these processes shown in Fig. 4d. First, B16-F10 cells were subcutaneously injected into the left back of mice aged 6–7 weeks to form the initial tumor; this was used for photothermal therapy. B16-F10 cells were then injected subcutaneously on the other side six days later to form metastatic tumors. When the initial tumor size was about 100 mm^3^, nine days after the first inoculation of tumor cells, aPD-L1 NVs-ICG was injected via the tail vein. On day ten, an 808 nm NIR laser (0.75 W/cm^2^, 8 min) was used to irradiate the initial tumor on the left side for photothermal treatment, and the NIR thermal imager was used to monitor the temperature change of tumor site in real time. The results show that tumors treated with aPD-L1 NVs-ICG after laser irradiation, plus the warming capacity of the melanoma itself, led to a surface temperature of 60 ℃ that could ablate the tumor (Fig. 4e). This implies that aPD-L1 NVs-ICG can better aggregate at the tumor site.

Changes in tumor volume were then closely measured to evaluate the therapeutic effects of different therapeutic regimens on tumors (Fig. 4f). The results showed that the left tumor was gradually ablated after the photothermal treatment with aPD-L1 NVs-ICG under laser radiation; tumor cell necrosis can be observed by HE staining (Additional file 1: Figure S4d). On the contrary, tumors in the NVs-ICG group and the free NVs group were inhibited only slightly. In this control, little photosensitizer accumulates at the site of the tumor, and there was no temperature change. the tumors in the aPD-L1 NVs group were not significantly inhibited indicating that even if the immunocheckpoint inhibition effect of aPD-L1 NVs might exist, the therapeutic effect was blocked.

Meanwhile, the contralateral side tumors without treatment were not significantly diminished in the aPD-L1 NVs-ICG photothermal treatment group (Additional file 1: Figure S4b). The results indicate that a single photothermal treatment could not inhibit the remote tumor even if the survival time of tumor-bearing mice increased tumor necrosis factor (TNF-α), inflammatory cytokines (IL-6, IL-12, IFN-γ), and immune cells (CD11c + CD86 + DCs and CD3a + CD8 + T cells) (Fig. 4g–i and Additional file 1: Fig. S5a–c). Notably, aPD-L1 NVs have the same function as aPD-L1, and can specifically bind to the PD-L1 ligand on the surface of tumor cells, so as to competitively inhibit the binding of the PD-1 receptor on the surface of immune cells, such as T lymphocytes, thus alleviating the inhibition ability of immune cell function by immune checkpoint and enhancing anti-tumor immunity. Moreover, the tumor's immune environment has been reconstituted by photothermal ablation, which may provide a benefit for immunotherapy to amplify tumor regression. Therefore, the nano-vaccine could activate the immune system to achieve a combined treatment that inhibits the growth of distant tumors.

### Preparation and characterization of the AAI

**Fig. 5:**
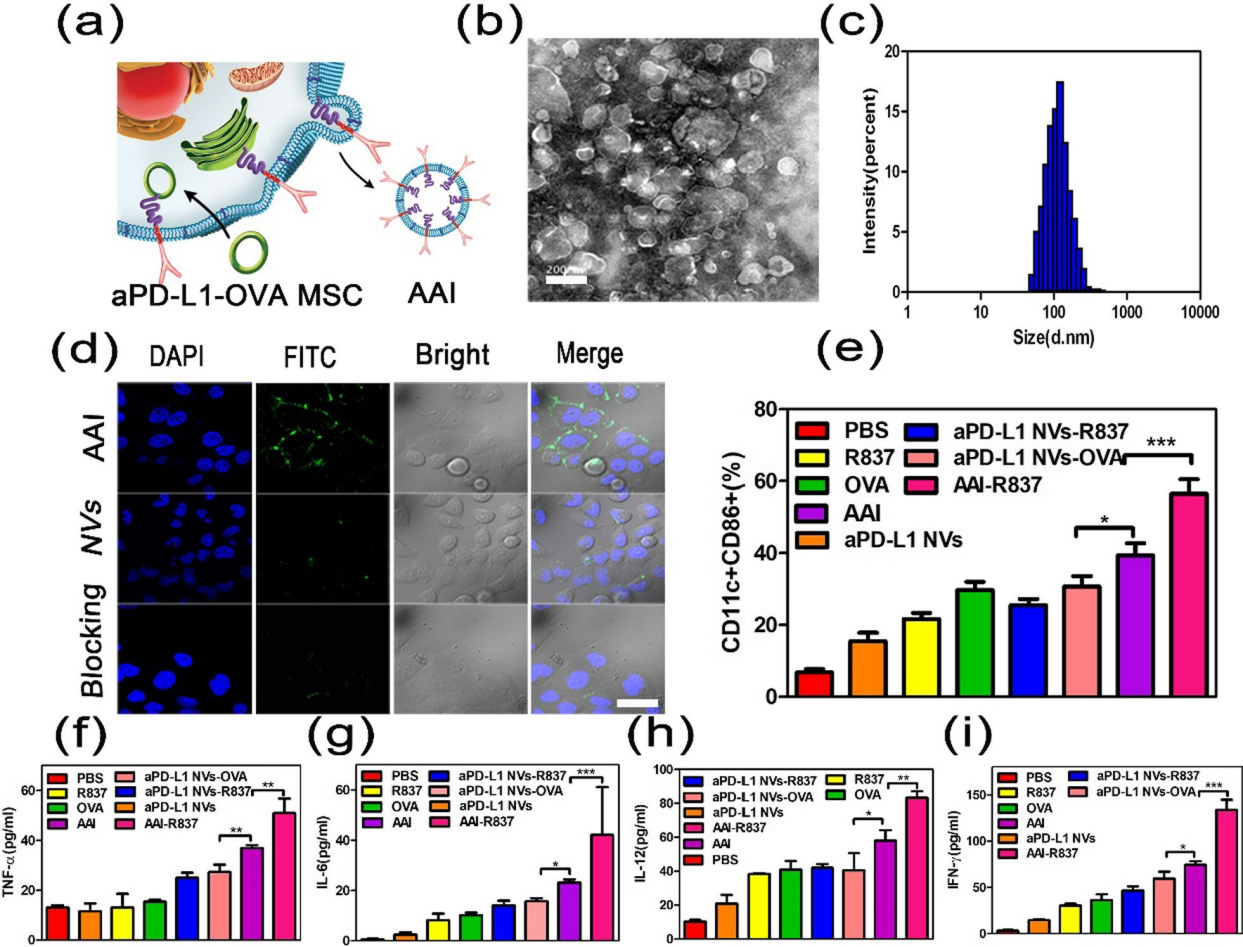
Preparation and characterization of the AAI. **a**Schematic diagram of aPD-L1 and OVA co-displayed MSC and AAI extraction. **b**TEM images for AAI negatively stained with uranyl acetate. Scale bar: 200 nm. **c** Dynamic diameter of aPD-L1 NVs determined by the dynamic light scattering (DLS). **d** CLSM image to show the ability of AAI to specifically target binding with DC2.4 cells. AAI were labeled with FITC and nuclei with DAPI. Scale bar: 50 µm. **e** The expression of CD11c and CD86 (markers for DC maturation) in BMDCs was detected by flow cytometry of after stimulated with each substance in vitro for 48 h. **f-i** The amount of TNF-α, IL-6, IL-12, IFN-γ in the DC 2.4 cell culture supernatant determined by ELISA after BMDCs incubation with different drugs for 48 h. P values were calculated by Student's t test (***P < 0.001, **P < 0.01 or *P < 0.05)

To acquire aPD-L1 and OVA co-displayed MSC, a plasmid comprising aPD-L1 and OVA fragments was constructed by combining aPD-L1 sequence and OVA protein sequences in tandem. MSCs were then infected via a lentiviral vector to make the aPD-L1 be displayed on the outside of the cell membrane and OVA antigen expressed on the inside of the cell membrane (Fig. 5a). The morphology and particle size of AAI were studied by TEM and DLS, respectively; the results showed that the morphology was the same as that of aPD-L1 NVs (Fig. 5b, c).

The ability of AAI to specifically target binding of DCs is the basis for the realization of lymph nodes in vivo. Therefore, we further verified the interaction between AAI with DCs in vitro first. The DC2.4 cells were cultured with FITC-labeled NVs and AAI for 3 h, respectively. Importantly, we observed AAI bound to DC2.4 cells with CLSM while free NVs showed a low DC2.4 cell membrane affinity (Fig. 5d). Furthermore, after adding aPD-L1 to the blocked PD-L1 receptor, the AAI no longer bound to the DC2.4 cell membrane. It follows that AAI can bind to DC2.4 cell membranes because of specific binding of the aPD-L1 to the PD-L1 receptor. To summarize, the NV displaying the aPD-L1 showed a specific double-targeting ability against cancer cells and APCs. AAI was used to load immunologic adjuvant imiquimod (R837, AAI-R837)—a toll-like-receptor-7 agonist, and the loading efficiency of R837 was further evaluated to be 20% via high performance liquid chromatography (HPLC) (Additional file 1: Figure S1b, c).

DCs are closely related to the occurrence and development of tumors. The core of an anti-tumor immune response is to generate a cellular immune response with CTLs as the main body; this is the basis of DCs in immunotherapy [42, 43]. Accordingly, nano-vaccine stimulates the maturation and differentiation of DCs and is a critical link to activate and enhance the immune effectiveness of the immune system in vivo [44–46]. Hence, we investigated the effect of AAI-R837 as a nanovaccine on bone marrow-derived DCs (BMDCs) separated from C57BL/6 mice in vitro.

After AAI-R837 was co-incubated with BMDCs for 48 h, the expression of mature biomarkers CD11c + CD86 + on BMDCs was analyzed by flow cytometry (FCM). The data indicated that the content of CD11c + CD86 + increased compared to PBS-treated groups (Fig. 5e). However, the effect of AAI to upregulate CD11c + CD86 + was significantly higher than that of free NVs and free OVA perhaps because AAI expressing TAA and aPD-L1 at the same time can better deliver the antigen to DCs and stimulate their maturation. Moreover, AAI-R837 triggered the highest level of CD11c + CD86 + expression relative to free R837, NVs-R837, and free OVA; its strongest stimulation ability was with DCs. These results show that AAI-R837 displaying aPD-L1 can specifically bind to BMDCs and can deliver the immunologic adjuvant and TAA to BMDCs, thus significantly enhancing their differentiation.

Furthermore, after being co-incubated for 48 h, the cell culture medium was collected, and the content of immune-related cytokines (IL-6, IL-12, TNF-α, and IFN-γ) was detected by ELSA (Fig. 5f–i). The content of these cytokines related to DC cell maturation was significantly increased versus other control groups; AAI-R837 further indicated superiority to promote DCs maturation.

### Biological distribution of AAI and photoimmunotherapy on tumor combines nanomissiles with nanovaccines in vivo

**Fig. 6:**
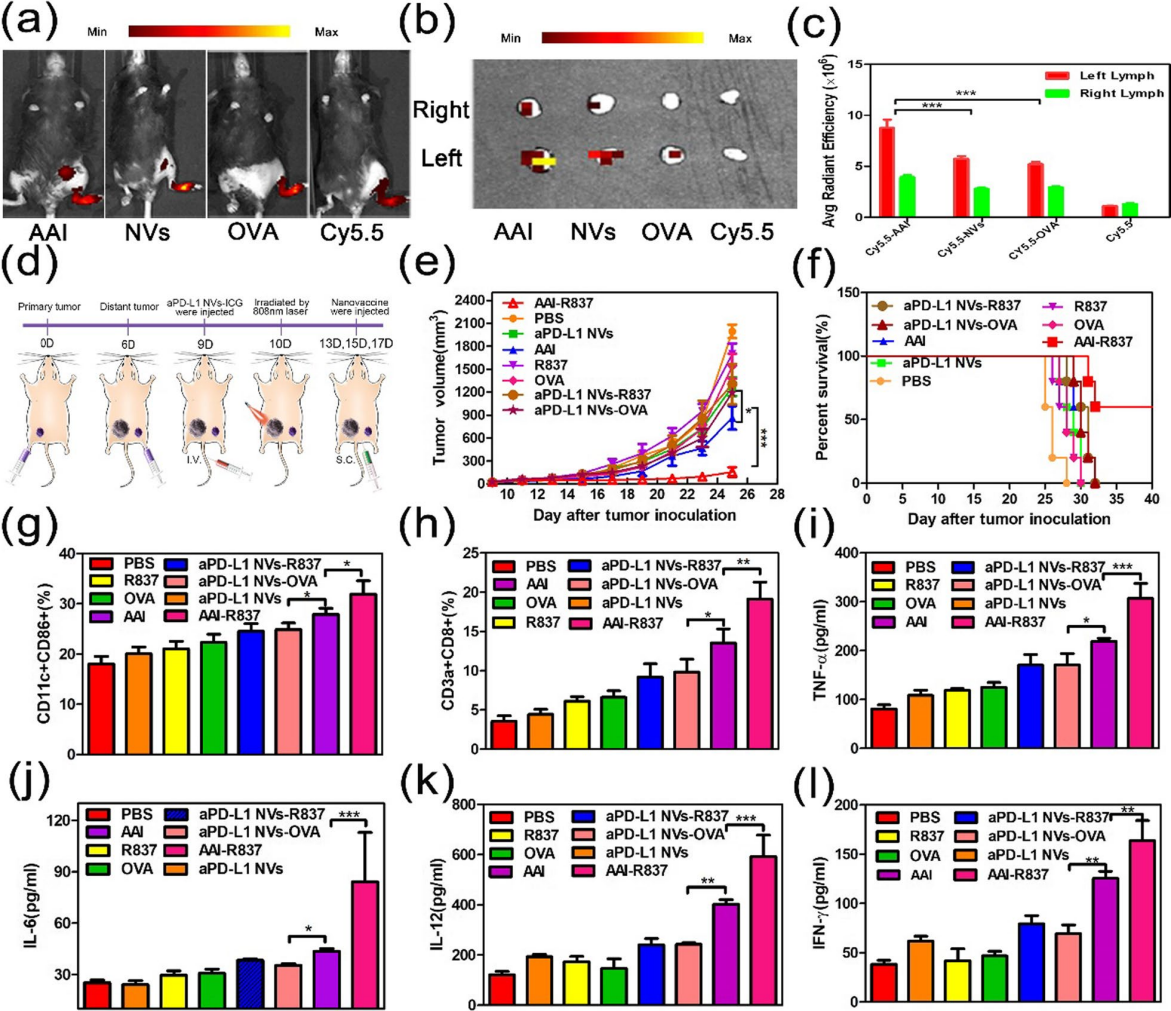
Biological distribution of AAI and photoimmunotherapy on tumor combine nanomissiles with and nanovaccines in vivo. **a** Fluorescent (FL) images of C57/BL-6 mice at 96 h after the drug was injected into the sole of the left foot. **b**, **c** FL images and fluorescence signal intensity of inguinal lymph nodes of the mice in **a**. **d** Schematic diagram of photothermal-immunotherapy for double tumor-bearing mice. **e** Tumor volume growth curve of distant secondary tumors after photoimmunotherapy with different drugs. **f** Survival curves of the mice after photoimmunotherapy with different drugs. **g** The content of mature DC cells in the lymph nodes of mice was detected via analysis of dendritic cell maturation markers (CD11c + CD86 +) by flow cytometry after last dose for 3 d. **h** The content of CD8 + T cells in distant secondary tumors of mice was detected via analysis of CD8 + T cells markers (CD3a + CD8 +) by flow cytometry after last dose for 3 d. **i-l** The amount of TNF-α, IL-6, IL-12, IFN-γ in serum of the mouse determined by ELISA after last dose for 3 d. n = 3 ~ 5. P values were calculated by Student's t test (***P < 0.001, **P < 0.01 or *P < 0.05)

Encouraged by the results in vitro, we further investigated the effect of AAI-R837 on the immune response in vivo. First, we verified the process of AAI uptake by DCs and migration to lymph nodes in vivo. AAI, free NVs, and OVA labeled with fluorescent dye Cy5.5 were injected subcutaneously into the right leg of mice, and the migration process of drugs in vivo was observed by an IVIS Lumina II imaging system. The injected free Cy5.5 and Cy5.5-OVA rapidly diffused and metabolized in the body although there was a certain concentration of inguinal lymph nodes in the early stage. The product was metabolized quickly over time (Fig. 6a). Cy5.5-NVs diffuses slowly in vivo, mainly accumulates at the injection site, and only slightly accumulates at the inguinal lymph node site. Although Cy5.5-AAI also diffused slowly, it gradually migrated to the inguinal lymph nodes in vivo and showed enrichment at 96 h. The left and right inguinal lymph nodes of all of the mice were collected, and the fluorescence signal of the lymph nodes was observed by an FL imaging system. The results show that the fluorescence signal from Cy5.5-AAI was significantly higher than that of others (Fig. 6b, c). These data confirmed that Cy5.5-AAI was specifically absorbed by DCs and migrated to lymph nodes in vivo.

Next, photoimmunotherapy was done on the tumor. After that, various immunological materials were injected subcutaneously at the root of the tail on 13, 15, and 17 d as a vaccine for immunotherapy on remnant tumors (Fig. 6d). After immunization with different substances, the growth of distal tumors and survival were closely monitored. In all groups, the nanovaccine(AAI-R837) had the best tumor inhibition (Fig. 6e and Additional file 1: Fig. S8a). It cured 60% of the mice with cancer (Fig. 6f). In addition, aPD-L1 NVs-837 and aPD-L1 NVs-OVA were more effective at inhibiting the growth of tumors than free R837 and OVA, and can prolong the survival time. Furthermore, tumor growth is slower when treated with AAI than aPD-L1 NVs-OVA perhaps because antigens expressed on cell membranes can better convey antigenic signals and promote the immune system's response versus OVA.

The immune effect after treatment was also studied. Three days after the final immunization, distal tumors, serum, and lymph nodes were collected, and immune cells and cytokines related to the immune response were detected. A cell suspension was prepared by homogenized the material, and the content of mature DCs in lymph nodes, as well as CTLs in tumor was analyzed by FCM (Fig. 6g, h). Cell flow data showed that the contents of mature DC cells as well as CTLs changed after the treatment with control drugs. Particularly, more CTLs were induced by aPD-L1 NVs-837 and aPD-L1 NVs-OVA were 1.5-fold higher than free R837 and OVA; AAI was 0.5 times higher than aPD-L1 NVs-OVA; and AAI-R837 was higher than AAI, which is consistent with the above treatment effect. TNF-α, IL-6, IL-12, and IFN-γ in serum were measured by ELSA (Fig. 6i-l). The change in the secretion of these cytokines was the same as changes in immune cells. These results showed that aPD-L1 NV could be used for targeted delivery, and AAI-R837 successfully enhanced the activation of the immune system to inhibit tumor growth without any adverse effects in the case of biopsy or weight gain (Additional file 1: Figure S4e, f and S8b, c).

## Conclusions

In summary, we constructed AAI that simultaneously express antibodies and tumor-related antigens using the cell membrane of bone marrow mesenchymal stem cells. Nano-vesicles expressing aPD-L1 can specifically bind to PD-L1 receptors on cancer cells and can be utilized to deliver photosensitizers to tumor sites for efficient photothermal therapy. Meanwhile, the aPD-L1 expressed on the modified nanocapsule can also specifically bind to the PD-L1 receptor on the DCs. In short, this design takes advantage of specific binding at the immune checkpoints. This provides a promising strategy for targeted drug delivery. Intriguingly, the immune environment in the tumor region was programmed via photothermal ablation. This led to improved immunotherapy to arrest the residual and distal tumor. Additionally, antibodies and antigens were co-expressed on AAI-R837 and delivered the immune adjuvant to the antigen presenting cells. Antigen signal was sent to the DCs correspondingly and the powerful immune response of the immune system was elicited. The experimental results indicated that the combined treatment of photoimmunotherapy on the melanoma model not only had a significant inhibiting effect on the initial tumor, but also inhibited distant tumor. The low immunogenicity of the materials extended the in vivo circulation time of the nanocarrier, and its biological safety suggests clinical applications.

## Materials and methods

### Materials

Imiquimod (R837) and Indooyanine gree (ICG) were purchased from Invivogen and BBI Life Science Corporation, respectively. Ovalbumin (OVA), calcein acetoxymethyl ester (calcein AM) and propidium iodide (PI) were purchased from Sigma-Aldrich Inc. FITC was purchased from MedChemExpress LLC and Cy5.5 was purchased from APExBIO Technology LLC. Anti-mouse CD274 (B7-H1, PD-L1), FITC-anti-mouse CD86, PE-anti-mouse CD11c, TITC-anti-mouse CD8 and PerCP-anti-mouse CD3ε for flow cytometry were purchased from Biolegend Inc. Basement Membrane Matrix was purchased from Becton, Dickinson and Company. TNF-α, IL-6, IL-12 and IFN-γ ELSA kit were purchased from Beyotime Biotech Inc.

## Methods

### Culture and transformation of mesenchymal stem cells

Bone marrow mesenchymal stem cells were extracted from the bone marrow of suckling C57BL/6 mice (Seven days after birthed) referring to the established method [47]. The mice were purchased from Shanghai slake laboratory animal Co. Ltd. C57BL/6 mouse bone marrow mesenchymal stem cell complete culture medium from Cyagen Biosciences Inc. was used for culturing MSCs at 37 °C under 5% CO_2_. To make aPD-L1 be expressed on membrane of MSCs, the aPD-L1 gene with a signal peptide, flag tags, aPD-L1 ScFv and transmembrane sequence were synthesized. While to make aPD-L1 and OVA be co-expressed on membrane of MSCs, plasmids was constructed containing signal peptide, flag tags, aPD-L1 ScFv, transmembrane sequence as well as an intramembrane fragment containing an OVA sequence.

1. DNA sequence of membrane-located signal peptide (from integrin beta 1):ATGAATTTGCAACTGGTTTCCTGGATTGGATTGATCAGTTTGATTTGTTCTGTATTTGGCCAAACAGATAAA
2. DNA sequence of Flag tag:
  GACTACAAGGACGACGACGACAAG
3. DNA sequence of aPD-L1 ScFv:
  GCCCAGGCCGCCCTGACCCAGCCCAGCAGCGTGAGCGCCAACCTGGGCGGCACCGTGAAGATCACCTGCAGCGGCGGCAGCGGCAGCTACGGCTGGTACCAGCAGAAGGCCCCCGGCAGCGCCCCCGTGAGCCTGATCTACGACAACACCAACAGGCCCAGCGACATCCCCAGCAGGTTCAGCGGCGCCCTGAGCGGCAGCACCGCCACCCTGACCATCACCGGCGTGCAGGCCGAGGACGAGGCCGTGTACTACTGCGGCAGCAGGGACAGCAGCAACGCCGGCAGCGTGTTCGGCGCCGGCACCACCCTGACCGTGCTGGGCCAGAGCAGCAGGAGCAGCGGCGGCGGCGGCAGCAGCGGCGGCGGCGGCAGCGCCCTGACCCTGGACGAGAGCGGCGGCGGCCTGCAGACCCCCGGCGGCGCCCTGAGCCTGGTGTGCAAGGCCAGCGGCTTCACCTTCAGCGACAGGGGCATGCACTGGGTGAGGCAGGCCCCCGGCAAGGGCCTGGAGTGGGTGGGCGCCATCAGCAGGAGGGGCAGCACCACCACCTACGCCCCCGCCGTGAAGGGCAGGGCCACCATCACCAGGGACAACGGCCAGAGCACCGTGAGGCTGCAGCTGAACAACCTGACCGCCGAGGACACCGCCACCTACTTCTGCGCCAAGAACGACGACAGCGTGGGCATCGTGACCACCAGCACCATCGACGCCTGGGGCCACGGCACCGAGGTGATCGTGAGCAGCACCAGCGGCCAGGCCGGCCAGCACCACCACCACCACCACGGCGCCTACCCCTACGACGTGCCCGACTACGCCAGC.
4. DNA sequence of transmembrane fragment:
  TTATGGGTCATCCTGCTGAGTGCTTTTGCCGGATTGTTGCTGTTAATGCTGCTCATTTTAGCACTGTGG
5. DNA sequence of OVA:
  ATGGGCTCCATCGGCGCAGCAAGCATGGAATTTTGTTTTGATGTATTCAAGGAGCTCAAAGTCCACCATGCCAATGAGAACATCTTCTACTGCCCCATTGCCATCATGTCAGCTCTAGCCATGGTATACCTGGGTGCAAAAGACAGCACCAGGACACAGATAAATAAGGTTGTTCGCTTTGATAAACTTCCAGGATTCGGAGACAGTATTGAAGCTCAGTGTGGCACATCTGTAAACGTTCACTCTTCACTTAGAGACATCCTCAACCAAATCACCAAACCAAATGATGTTTATTCGTTCAGCCTTGCCAGTAGACTTTATGCTGAAGAGAGATACCCAATCCTGCCAGAATACTTGCAGTGTGTGAAGGAACTGTATAGAGGAGGCTTGGAACCTATCAACTTTCAAACAGCTGCAGATCAAGCCAGAGAGCTCATCAATTCCTGGGTAGAAAGTCAGACAAATGGAATTATCAGAAATGTCCTTCAGCCAAGCTCCGTGGATTCTCAAACTGCAATGGTTCTGGTTAATGCCATTGTCTTCAAAGGACTGTGGGAGAAAGCATTTAAGGATGAAGACACACAAGCAATGCCTTTCAGAGTGACTGAGCAAGAAAGCAAACCTGTGCAGATGATGTACCAGATTGGTTTATTTAG.
  AGTGGCATCAATGGCTTCTGAGAAAATGAAGATCCTGGAGCTTCCATTTGCCAGTGGGACAATGAGCATGTTGGTGCTGTTGCCTGATGAAGTCTCAGGCCTTGAGCAGCTTGAGAGTATAATCAACTTTGAAAAACTGACTGAATGGACCAGTTCTAATGTTATGGAAGAGAGGAAGATCAAAGTGTACTTACCTCGCATGAAGATGGAGGAAAAATACAACCTCACATCTGTCTTAATGGCTATGGGCATTACTGACGTGTTTAGCTCTTCAGCCAATCTGTCTGGCATCTCCTCAGCAGAGAGCCTGAAGATATCTCAAGCTGTCCATGCAGCACATGCAGAAATCAATGAAGCAGGCAGAGAGGTGGTAGGGTCAGCAGAGGCTGGAGTGGATGCTGCAAGCGTCTCTGAAGAATTTAGGGCTGACCATCCATTCCTCTTCTGTATCAAGCACATCGCAACCAACGCCGTTCTCTTCTTTGGCAGATGTGTTTCCCCTTAA.

Then, Lentivirus vector encoding corresponding sequence was applied to infect MSCs. Infected MSCs displaying aPD-L1 with or without OVA were cultured in C57BL/6 mouse bone marrow mesenchymal stem cell complete culture medium maintained in 20% FBS and 1% Penicillin and streptomycin.

### Preparation of cell membrane nanovesicles

In the preparation of nanovesicles, when the number of cells reaches 5╳10^6^ in a petri dish, the medium in the culture dish with MSCs was removed, then the MSCs were scraped away by cell scraping, and were collected into the centrifuge tube with phosphate buffer solution (PBS). After 5% deoxysodium cholate, a surfactant, was added to the cell suspension (v:v = 1:100), MSCs were broken by a low power ultrasound (20 w, 4 ℃) for 10 to 20 s using the ultrasonic crusher (Sonics&Materials Inc.), and the protease inhibitor PMSF (10 mg/mL) was added (v:v = 1:200) to worked solution immediately. To remove the cytoplasm and nucleus, the suspending liquid was centrifuged at 3500 rpm (4 ℃) for 5 min. Supernatant was collected for further centrifugation at 15,000 rpm(4 ℃) for 20 min to acquire nanoscale membrane vesicles, then resulting vesicles were quantified by BCA kit (Beyotime Biotech Inc.) and dissolved in PBS and stored at 4 ℃ for standby application.

### Drug loading

After 200 μL ICG aqueous solution (1 mg/mL) was mixed with NVs suspension come from 5×10^6^ MSCs, the ultrasonic crushing instrument (20 w) was used for ultrasonic concussion for three times at 4 ℃, each session lasts 5 to 10 s and interrupted at least 10 s. After resulting sample was centrifuged at 12,000 rpm (4 ℃) for 20 min, the content of ICG in the supernatant was detected by a microplate analyzer, and the loading efficiency of ICG was calculated. NVs loading drugs were dispersed in PBS and stored at 4℃ for further study. When loading the immune adjuvant, 100 μL R837 (0.5 mg/mL) was added to NVs suspension, and the loading was carried out according to the same method mentioned above. The loading efficiency of R837 was determined by HPLC (Waters 1525)-UV visible detector at 325 nm. Acetonitrile was used as the mobile phase. ICG encapsulation and the UV absorption spectra of NVs-ICG were measured by microplate reader.

### Detection of characterization

The expression of aPD-L1 on the MSCs membrane was verified by immunofluorescence. The morphology and structure of NVs were characterized by fetenay transmission electron microscopy (F20). The dynamic particle size and electric potential of NVs was measured with Zeta sizer Nano-ZS (Malvern Instruments). The existence of aPD-L1 on the NVs was detected with coimmunoprecipitation (CO-IP) assay and Western blot (WB) analysis. In order to study photothermal performance of acquired materials, aPD-L1 NVs-ICG and ICG with different concentrations were dispersed in ultrapure water, then the samples were irradiated with 808 nm laser (1 W/cm^2^) for 5 min. During the heating of each solution, an NIR thermal imaging camera was used to record the temperature changes.

### Nanovesicles cell binding assay

To exam the ability of aPD-L1 NVs to binding to tumor cells, B16F10 cells were seeded in confocal culture dish (3 × 10^4^ cells/well) and cultured for 24 h. FITC-labeled aPD-L1 NVs or NVs were incubated with the cancer cells for 3 h, and cells in the other group were incubated with antiPD-L1 antibodies (10 μg/mL) for 2 h before the addition of aPD-L1 NVs to the culture medium. The NVs that were not bound to the cells were washed with PBS for three times, then the nuclei were stained with DAPI for 15 min. After the excess dye was washed and added new medium to the wells, confocal microscopy was performed under a confocal laser scanning microscope (LSM780) with 60 × oil-immersion lenses. The binding of AAI to DC2.4 cells was observed by the same method.

### Cytotoxicity of aPD-L1 NVs-ICG and AAI-R837

DC2.4 cells and B16F10 cells were selected to determine the cytotoxicity of the nanomedicine by standard MTT assay. DC2.4 cells and B16F10 cells were cultured in 96-well plates (1 × 10^4^ cells/well) for 24 h, respectively. After the cells were washed with PBS for three times, fresh medium containing different concentrations of aPD-L1 NVs-ICG and AAI-R837 was added for training another 24 h. The cell relative viability was detected by MTT assay and the cytotoxicity of the nanomedicine was determined.

### In vitro photothermal therapy of B16 cells with aPD-L1 NVs –ICG

To study the effect of photothermal therapy on B16F10 Cells with aPD-L1 NVs-ICG, B16F10 cells were cultured in 96-well plates(1 × 10^4^ cells/well) for 24 h. After washing the cells with PBS for three times, fresh medium containing 100 μg/mL of aPD-L1 NVs-ICG was added. Treated cells were irradiated with 808 nm laser (1.0 W/cm^2^) for 5 min. Viability of B16F10 cell was then assessed using standard MTT procedure. In order to intuitively evaluate the relative viability of B16F10 cells after different treatments, 2 μmol/L calcein acetoxymethyl ester (calcein AM) and 4 μ mol/L propidium iodide (PI) were used to stain the living and dead cells for 15 min, respectively. After washed with PBS, the cells were observed with inverted fluorescence microscope (Nikon, Japan).

### In vitro dendritic cell activation

Bone marrow derived dendritic cells (BMDCs)were extracted from the bone marrow of 6 ~ 8 weeks old C57BL/6 mice according to the established method [48]. BMDCs were cultured with RPMI-1640 containing 10% FBS and 1% streptomycin-penicillin in conventional environment. When enough BMDCs are present, the medium was changed to a new medium containing different immune drugs at equivalent 2 μg of OVA or 3 μg of R837 or approximately 10 μg of vesicular proteins and cultured for 48 h. Supernatant was collected and the presence of proinflammatory cytokines was detected applying the mouse TNF-α, IL-6, IL-12 and IFN-γ ELISA kit according to the manufacturer's instructions. DCs after different treatments were collected with PBS, and incubated with TITC-anti mouse-CD11c and PE-anti mouse-CD86 at a dilution ratio of 1:200 for 30 min in room temperature. After excessive antibodies were removed, the cellular fluorescence was detected by FACSC (Beckman Gallios). The flow data was analyzed with FlowJoV10 software.

### Biodistribution

50 μg of ICG, 100 μg of NVs-ICG and aPD-L1 NVs-ICG (containing 50 μg of ICG) dispersed in 100 μL PBS were injected into subcutaneous melanoma burdened C57BL/6 mice via tail vein. Fluorescence imaging system (IVIS Lumina II imaging system, Xenogen, USA) was used to obtain fluorescence images at 0, 6, 12, 24 and 48 h after injection, respectively. At 48 h, the main organs and tumors of the mice were collected, and fluorescence imaging and intensity records were collected.

### Detection of subcutaneous migration

NVs, OVA and AAI were labeled with Cy5.5, subsequently, and 50 μL normal saline with equivalent 10 μg of OVA or approximately 50 μg of vesicular proteins injected into unilateral foot pad of C57BL/6 mice. Fluorescence imaging in vivo was obtained carrying out the system of fluorescence images at different time points (0, 3, 6, 12, 24, 48, 72 and 96 h). The left and right inguinal lymph nodes of all mice were collected at 96 h after injected, fluorescence images were obtained and the fluorescence signal intensity was calculated using the imaging system.

### Photoimmunotherapy on tumor combine nanomissiles with and nanovaccines in vivo

Female C57BL/6 mice (6 ~ 8 weeks) were purchased from Shanghai slake laboratory animal co., LTD and used according to the agreement approved by committee of Xiamen University Laboratory Animal Center. The mice were randomly divided into groups. 3 × 10^6^ OVA-expressing B16F10 cells were suspended in PBS and subcutaneously injected into the left posterior back of C57BL/6 mice to challenged primary tumor. On 6 d, 3 × 10^6^ OVA-expressing B16F10 cells were subcutaneously injected into the right posterior back of C57BL/6 mice to challenged distant tumor. On 9 d after first inoculation, primary tumor was about 200 mm^3^ in size, various formulations were injected into the tail vein containing equivalent 4.5 mg/kg of ICG or approximately 100 μg of vesicular proteins (five mice per group). After 12 h, the primary tumor was irradiated by 808 nm laser (0.75 W/cm^2^) for 8 min, and the temperature of tumor surface was monitored by near-infrared thermal imaging instrument. On 13, 15 and 17 d after challenge of the first tumor, mice treated with photothermal therapy were subcutaneously injected with different immune drugs containing equivalent 10 μg of OVA or 10 μg of R837 or approximately 50 μg of vesicular proteins for immunotherapy of distant tumor (five mice per group). The body weight of the mice was recorded using electronic balance, and the tumor size and volume were measured using a vernier caliper every other day. The calculation formula (V) was V = d^2^*D/2, d and D was mm in the shortest and longest diameters of the tumors respectively. Animals were euthanized when they showed signs of impaired health or tumors larger than 2cm^3^.

### In vivo immune response analysis

To test the immune response in the treated mice, the mice were sacrificed and serum, tumor, and lymph nodes were collected on 3 d after photothermal treatment or final immunization. The levels of TNF-α, IL-6, IL-12 and IFN-γ in serum were detected by ELISA kit according to the instructions. The tissues were homogenized to prepare single-cell suspension, and tumor cells were labeled with anti-mouse CD3a and CD8 antibodies, and lymph node cells were labeled with anti-mouse CD11c and CD86 antibodies. Then, the content of CD8 + T lymphocytes and mature DC cells were detected by flow cytometry.

### H&E staining

The main organs (liver, spleen, kidney, heart, and lung) and tumor tissues of mice in each treatment group were collected and fixed in 4% paraformaldehyde. Hematoxylin and eosin staining were performed before electron microscopy.

### Statistical analysis

All the statistical figures are mean ± standard deviation, P values were calculated by Student's t test (***P < 0.001, **P < 0.01 or *P < 0.05). All statistical analyses were performed using the IBM SPSS statistics 20.

## Supplementary Information

**Additional file 1: Figure S1.** (a) The standard curve of R837 was detected by HPLC; (b) The standard curve of ICG was measured by ultraviolet spectrophotometer; (c) Analysis of drug loading efficiency using nanometer delivery platform. **Figure S2.** The temperature change curve of the aPD-L1 NVs-ICG and ICG with corresponding concentration under the near infrared (NIR) laser irradiation at 808 nm wavelength (1 w/cm^2^, 5 min). **Figure S2.** The temperature change curve of the aPD-L1 NVs-ICG and ICG with corresponding concentration under the near infrared (NIR) laser irradiation at 808 nm wavelength (1 w/cm^2^, 5 min). **Figure S4. (a)** Temperature change curve of the tumor under different treatments under 808 nm laser irradiation (0.75 W/cm^2^, 8 min). **(b)**Tumor volume growth curve for irradiated distant secondary tumors (in right flank) in mice after PTT with different drugs. **(c)** Survival curves of the mice after PTT with different drugs. Images of Hematoxylin and eosin (H&E) staining of irradiated primary tumors and distant secondary tumors sections after various treatments, respectively. Scale bar: 100 µm. **(b)**Weight curve after PTT with the different substances. Images of H&E staining of the main viscera after PTT, respectively. Scale bar: 100 µm. **Figure S5.** Productions of IL-6 **(a)**, IL-12 **(b)**, TNF-α **(c)** in serum from the mouse determined by ELISA after treated with PTT for 3 d. P values were calculated by Student's t test (***P < 0.001, **P < 0.01 or *P < 0.05). **Figure S6.** Cell viability of the different concentrations of AAI-R837 on B16 cells and DCs after co-incubation for 24 h. **Figure S7.** The curve of fluorescence signal intensity in inguinal lymph nodes with time after subcutaneous injection of various drugs. **Figure S8. (a)** Images of Hematoxylin and eosin (H&E) staining of distant secondary tumors sections after photoimmunotherapy, respectively. Scale bar: 100 µm. **(b)** Weight curve after photoimmunotherapy with the different substances. **(c)** Images of H&E staining of the main viscera after photoimmunotherapy, respectively. Scale bar: 100 µm.

## Data Availability

The data are available in the main manuscript, additional information files, and from the corresponding authors upon reasonable request.
