# Supplementary material for: Functional nanovesicles displaying anti-PD-L1 antibodies for programmed photoimmunotherapy
Source: J Nanobiotechnology. 2022 Feb 2;20:61. doi: 10.1186/s12951-022-01266-3 (PMC8811970; doi:10.1186/s12951-022-01266-3)
Supplement: Supplementary file 1 — Additional file 1: Figure S1. (a) The standard curve of R837 was detected by HPLC; (b) The standard curve of ICG was measured by ultraviolet spectrophotometer; (c) Analysis of drug loading efficiency using nanometer delivery platform. Figure S2. The temperature change curve of the aPD-L1 NVs-ICG and ICG with corresponding concentration under the near infrared (NIR) laser irradiation at 808 nm wavelength (1 w/cm2, 5 min). Figure S2. The temperature change curve of the aPD-L1 NVs-ICG and ICG with corresponding concentration under the near infrared (NIR) laser irradiation at 808 nm wavelength (1 w/cm2, 5 min). Figure S4. (a) Temperature change curve of the tumor under different treatments under 808 nm laser irradiation (0.75 W/cm2, 8 min). (b)Tumor volume growth curve for irradiated distant secondary tumors (in right flank) in mice after PTT with different drugs. (c) Survival curves of the mice after PTT with different drugs. Images of Hematoxylin and eosin (H&E) staining of irradiated primary tumors and distant secondary tumors sections after various treatments, respectively. Scale bar: 100 µm. (b)Weight curve after PTT with the different substances. Images of H&E staining of the main viscera after PTT, respectively. Scale bar: 100 µm. Figure S5. Productions of IL-6 (a), IL-12 (b), TNF-α (c) in serum from the mouse determined by ELISA after treated with PTT for 3 d. P values were calculated by Student's t test (***P < 0.001, **P < 0.01 or *P < 0.05). Figure S6. Cell viability of the different concentrations of AAI-R837 on B16 cells and DCs after co-incubation for 24 h. Figure S7. The curve of fluorescence signal intensity in inguinal lymph nodes with time after subcutaneous injection of various drugs. Figure S8. (a) Images of Hematoxylin and eosin (H&E) staining of distant secondary tumors sections after photoimmunotherapy, respectively. Scale bar: 100 µm. (b) Weight curve after photoimmunotherapy with the different substances. (c) Images of H&E staining of the main v [file 12951_2022_1266_MOESM1_ESM.docx]

**Additional Material for**

**Functional Nanovesicles Displaying Anti-PD-L1 Antibodies for Programmed Photoimmunotherapy**

Hu Chen**^1,†^**, Pengfei Zhang**^1,2,†^**, Yesi Shi**^1^**, Chao Liu**^1^**, Qianqian Zhou**^3^**, Yun Zeng**^1^**, Hongwei Cheng**^1^**, Qixuan Dai**^1^**, Xing Gao**^1^**, Xiaoyong Wang**^1,*^**, and Gang Liu**^1,*^**

**^1^**State Key Laboratory of Molecular Vaccinology and Molecular Diagnostics & Center for Molecular Imaging and Translational Medicine, School of Public Health, Xiamen University, Xiamen 361102, China.

**^2^**Institute of Molecular Immunology, School of Laboratory Medicine and Biotechnology, Southern Medical University, Guangzhou 510080, China.

**^3^**Tongren Hospital, Shanghai Jiao Tong University School of Medicine, Shanghai, 200336, China.

*****Correspondence author, Email: [gangliu.cmitm@xmu.edu.cn](mailto:gangliu.cmitm@xmu.edu.cn); wangxy@xmu.edu.cn.

**^†^** These authors contributed equally to this work.


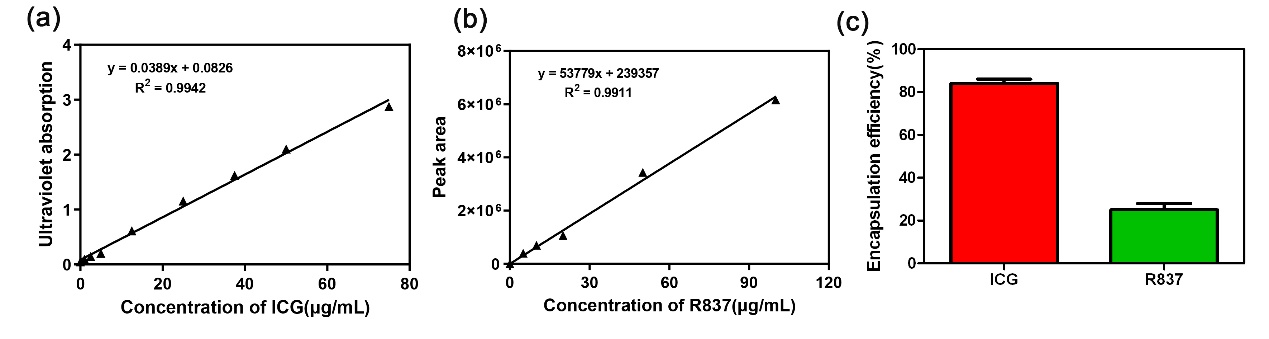


**Figure S1. (a)** The standard curve of R837 was detected by HPLC; **(b)** The standard curve of ICG was measured by ultraviolet spectrophotometer; **(c)** Analysis of drug loading efficiency using nanometer delivery platform.


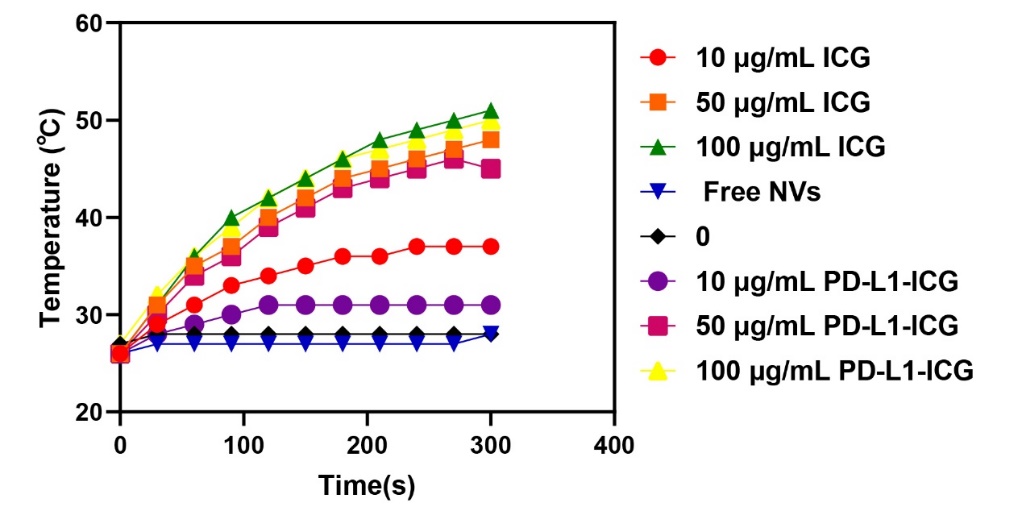


**Figure S2.** The temperature change curve of the aPD-L1 NVs-ICG and ICG with corresponding concentration under the near infrared (NIR) laser irradiation at 808nm wavelength (1 W/cm^2^, 5 min).


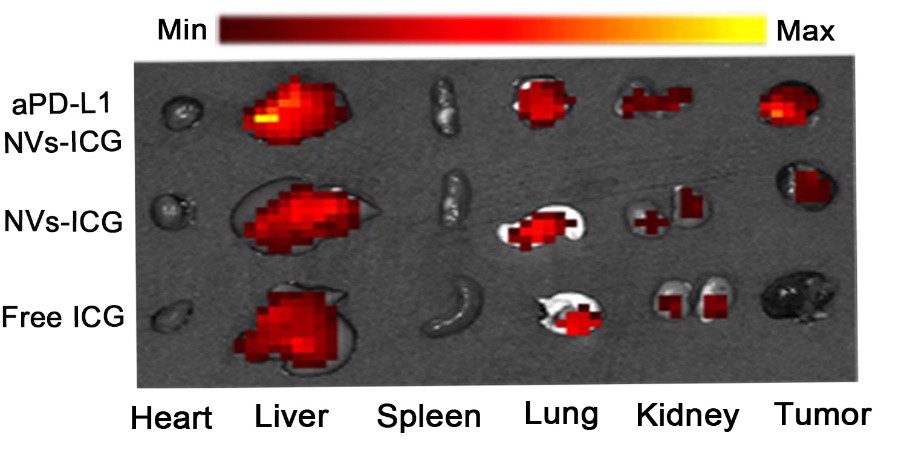


**Figure S3.** FL images of major organs and tumors of the mice in **Figure 4a** at 48 h after treatment.


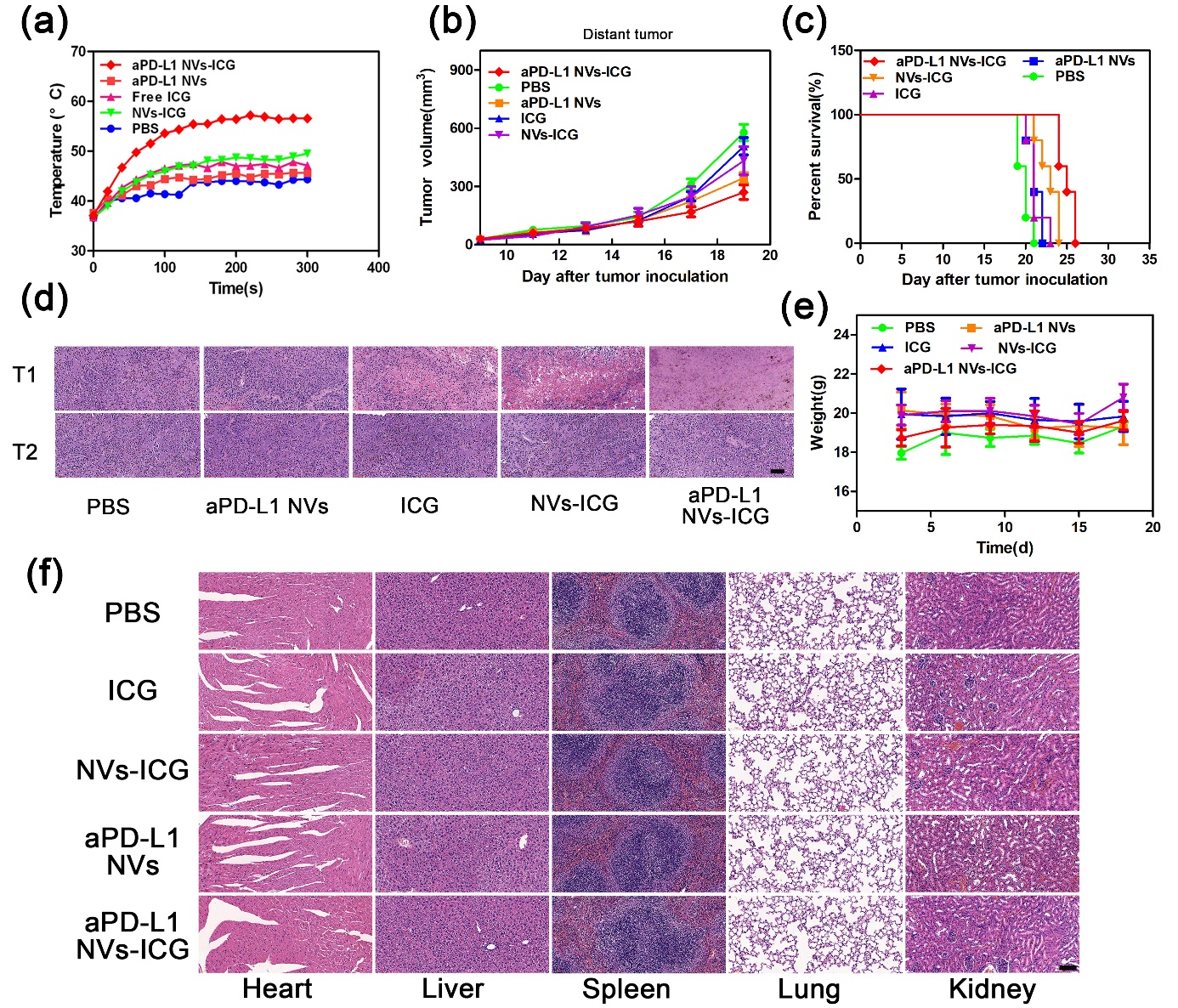
 **Figure S4. (a)** Temperature change curve of the tumor under different treatments under 808 nm laser irradiation (0.75 W/cm^2^, 8 min). **(b)** Tumor volume growth curve for irradiated distant secondary tumors (in right flank) in mice after PTT with different drugs. **(c)** Survival curves of the mice after PTT with different drugs. Images of Hematoxylin and eosin (H&E) staining of irradiated primary tumors and distant secondary tumors sections after various treatments, respectively. Scale bar: 100 µm. **(b)** Weight curve after PTT with the different substances. Images of H&E staining of the main viscera after PTT, respectively. Scale bar: 100 µm.


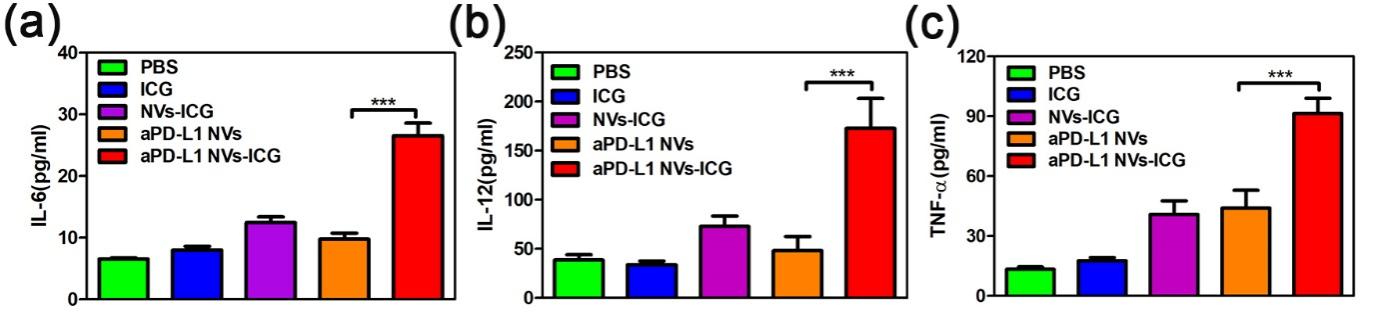


**Figure S5.** Productions of IL-6 **(a)**, IL-12 **(b)**, TNF-α **(c)** in serum from the mouse determined by ELISA after treated with PTT for 3 d. P values were calculated by Student's t test (***P < 0.001, **P < 0.01 or *P < 0.05).


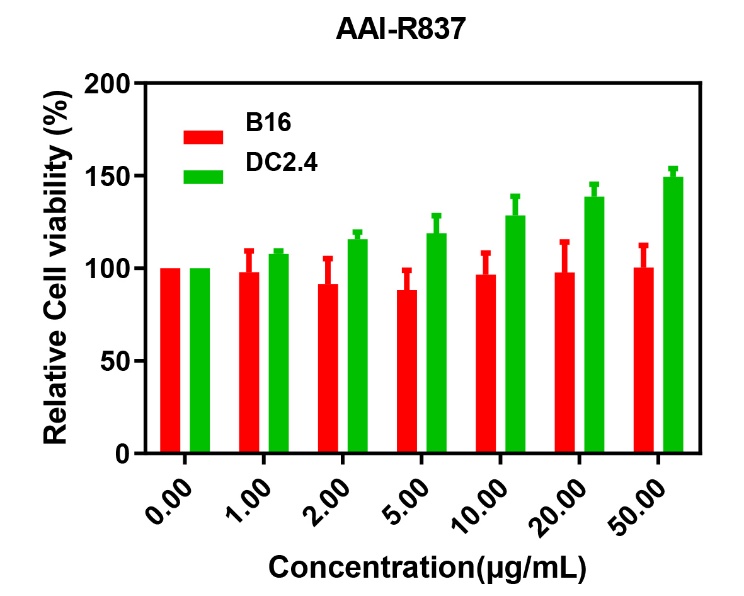


**Figure S6.** Cell viability of the different concentrations of AAI-R837 on B16 cells and DCs after co-incubation for 24 h.


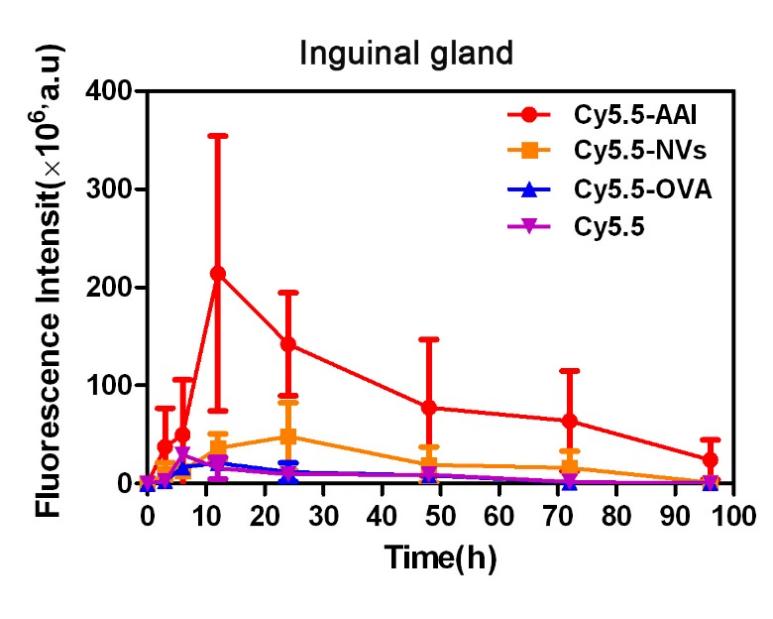


**Figure S7.** The curve of fluorescence signal intensity in inguinal lymph nodes with time after subcutaneous injection of various drugs.


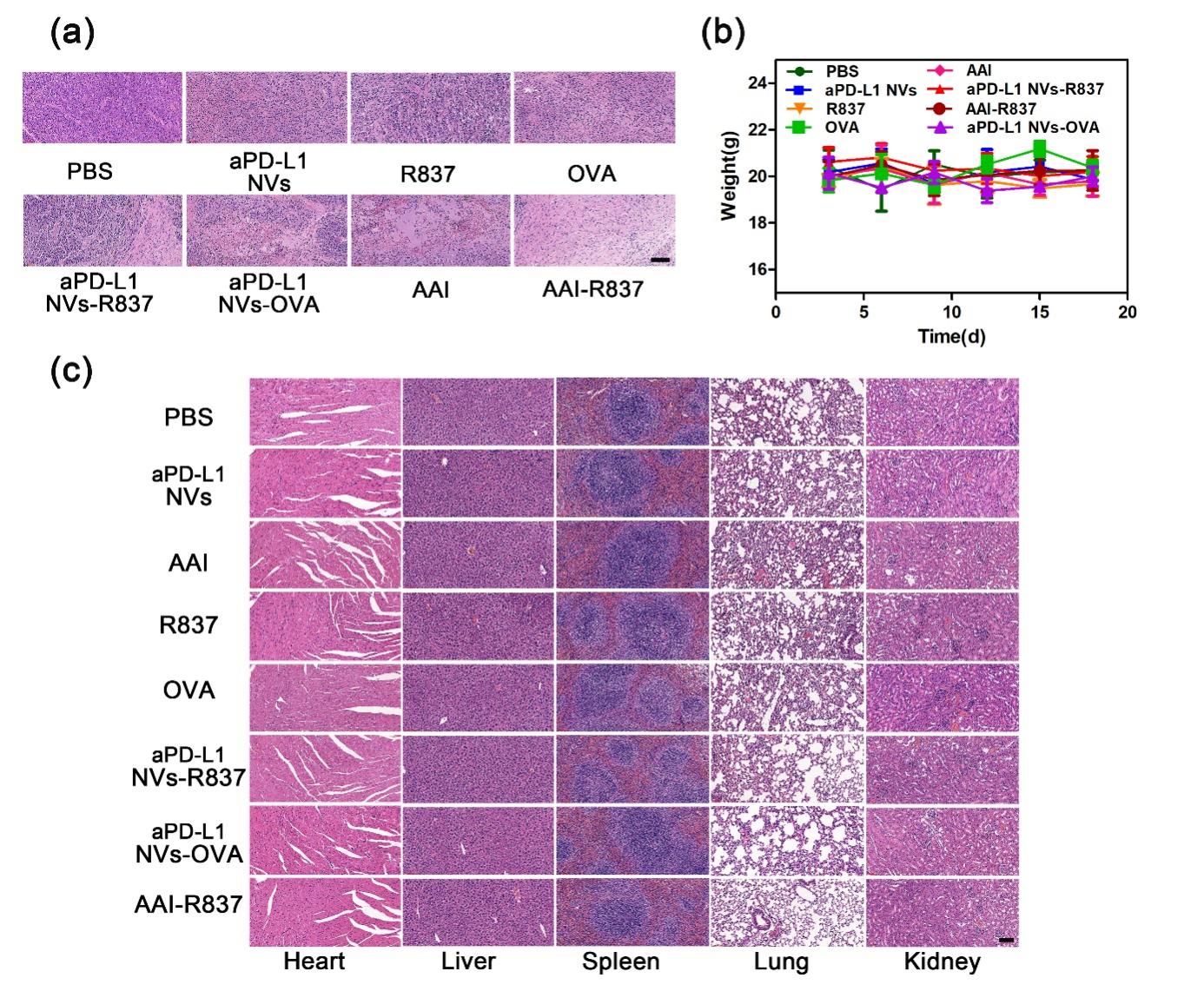


**Figure S8. (a)** Images of Hematoxylin and eosin (H&E) staining of distant secondary tumors sections after photothermal-immunotherapy, respectively. Scale bar: 100 µm. **(b)** Weight curve after photothermal-immunotherapy with the different substances. **(c)** Images of H&E staining of the main viscera after photothermal-immunotherapy, respectively. Scale bar: 100 µm.
